# Increased elasticity of sucrose demand during hyperdopaminergic states in rats

**DOI:** 10.1007/s00213-022-06068-x

**Published:** 2022-01-31

**Authors:** A. Maryse Minnaard, Mieneke C. M. Luijendijk, Annemarie M. Baars, Lisa Drost, Geert M. J. Ramakers, Roger A. H. Adan, Heidi M. B. Lesscher, Louk J. M. J. Vanderschuren

**Affiliations:** 1 Department of Population Health Sciences, Unit Animals in Science and Society, Faculty of Veterinary Medicine, Utrecht University, Utrecht, the Netherlands; 2 Department of Translational Neuroscience, University Medical Center Utrecht Brain Center, Utrecht University, Utrecht, the Netherlands

**Keywords:** Behavioural economics,, Chemogenetics,, Demand,, Dopamine,, Motivation,, Rats,, Ventral tegmental area

## Abstract

**Rationale:**

Deficits in cost–benefit decision-making are a core feature of several psychiatric disorders, including substance addiction, eating disorders and bipolar disorder. Mesocorticolimbic dopamine signalling has been implicated in various processes related to cognition and reward, but its precise role in reward valuation and cost–benefit trade-off decisions remains incompletely understood.

**Objectives:**

We assessed the role of mesocorticolimbic dopamine signalling in the relationship between price and consumption of sucrose, to better understand its role in cost–benefit decisions.

**Methods:**

Dopamine neurons in the ventral tegmental area (VTA) were chemogenetically activated in rats, and a behavioural economics approach was used to quantify the relationship between price and consumption of sucrose. Motivation for sucrose was also assessed under a progressive ratio (PR) schedule of reinforcement. To further gauge the role of dopamine in cost–benefit trade-offs for sucrose, the effects of treatment with D-amphetamine and the dopamine receptor antagonist alpha-flupentixol were assessed.

**Results:**

Chemogenetic activation of VTA dopamine neurons increased demand elasticity, while responding for sucrose under a PR schedule of reinforcement was augmented upon stimulation of VTA dopamine neurons. Treatment with amphetamine partially replicated the effects of chemogenetic dopamine neuron activation, whereas treatment with alpha-flupentixol reduced free consumption of sucrose and had mixed effects on demand elasticity.

**Conclusions:**

Stimulation of mesocorticolimbic dopaminergic neurotransmission altered cost–benefit trade-offs in a complex manner. It reduced the essential value of palatable food, increased incentive motivation and left free consumption unaltered. Together, these findings imply that mesocorticolimbic dopamine signalling differentially influences distinct components of cost expenditure processes aimed at obtaining rewards.

**Supplementary Information:**

The online version contains supplementary material available at 10.1007/s00213-022-06068-x.

## Introduction

Every day, we are confronted with situations requiring judgements and decisions. In fact, the ability to make decisions on the basis of costs and benefits can be considered a cornerstone of adaptive behaviour. Value-based decision-making entails a process in which humans and animals choose between competing courses of action by assessing the expected costs and the relative outcome values of each choice (Rangel et al. 2008; Tang et al. 2016). Deficits in cost–benefit trade-offs are a core feature of several psychiatric disorders, including substance addiction, eating disorders and bipolar disorder (American Psychiatric Association 2013; Cáceda et al. 2014; Goschke 2014). Therefore, a better understanding of the neurobiological mechanisms underlying cost–benefit trade-off decisions will provide more insight into these pathologies.

Derived from the field of behavioural economics, operant-based methods have been developed to study the relationship between price and consumption of goods (i.e. cost and benefit). Behavioural economics concentrates on the consumption of a commodity as a fundamental index of demand (Hursh et al. 2005), based on consumer demand theory, which considers how consumption varies as a function of price (Hursh 1980; Hursh et al. 1988). In rodent studies, the number of operant responses required for reinforcement is considered the price. The relationship between the consumption of a reinforcer and its price can then be described by a demand curve, which generally follows the law of demand: as price increases, consumption decreases (Hursh et al. 1988; Watson and Holman 1977).

Behavioural economics analysis offers insightful measures derived from operant self-administration data: (1) demand elasticity (signified by $\alpha$), which is the degree to which consumption decreases as price increases, and (2) demand intensity (signified by ${Q}_{0}$), which is the consumption at a minimally constrained price (Hursh and Silberberg 2008). Thus, demand elasticity reflects the degree to which the number of earned rewards decreases as the required effort per reward increases, while demand intensity reflects the hypothetical number of rewards an animal would consume if the response requirement was zero. Demand for a commodity is considered elastic when it decreases in response to proportionately small increases in price, whereas an inelastic demand reflects low sensitivity to price changes. The distinction between elastic and inelastic demand is a continuum since consumption of all goods eventually declines if the price is sufficiently elevated (Hursh et al. 2005). Because the price for a given good varies with elasticity changes, value cannot be pinpointed to one particular price. Therefore, to represent the scaled value of a reward, the term “essential value” is used in this manuscript, which is reflected by the rate of change in demand elasticity (Hursh and Silberberg, 2008). Moreover, the value of $\alpha$ also reflects the strength of a reinforcer as it is inversely related to the essential value (Hursh and Silberberg 2008). That is, reinforcers with a steep declining demand curve have a higher $\alpha$ value and thus a lower essential value than reinforcers with an inelastic demand curve that declines slowly when price increases. The measure of demand elasticity is thought to go beyond a response rate-based measure by more fully characterising the relationship between the price of a reinforcer and its consumption, as well as the underlying neural mechanisms.

A different operant-based method to study the exertion of effort for appetitive rewards is the progressive ratio (PR) schedule of reinforcement (Hodos 1961), which has been extensively used over the years to test the degree to which an animal maintains operant responding for reward as the response requirement increases after each reward delivery. The number of obtained rewards, lever presses and the highest ratio achieved (i.e. breakpoint) in PR tasks are common measures that are thought to reflect incentive motivation (Richardson and Roberts 1996; Salamone and Correa 2012).

The mesocorticolimbic dopamine system has been widely implicated in reward-directed behaviour, specifically in processes such as behavioural activation, salience, reward prediction error signalling and incentive motivation (Berridge and Robinson 1998; Bromberg-Martin et al. 2010; Hamid et al. 2016; Keiflin and Janak 2015; Robbins and Everitt 2007; Salamone and Correa 2012; Schultz, 2016). The mesocorticolimbic dopamine system comprises dopaminergic neurons originating in the ventral tegmental area (VTA), projecting to the nucleus accumbens and the prefrontal cortex. Especially dopamine signalling in the nucleus accumbens is thought to contribute to effort-based choice behaviour (Floresco 2015; Floresco et al. 2008; Mai et al. 2012; Salamone et al. 2016a, 2016b). This has been shown, for instance, in studies where administration of dopamine D1 and D2 receptor antagonists reduced breakpoints under a PR schedule of reinforcement in rats (Randall et al. 2012, 2014). Nucleus accumbens dopamine depletions have also been shown to make rats more sensitive to the effort requirements under different high ratio schedules (Aberman and Salamone 1999; Ishiwari et al. 2004). Conversely, stimulation of forebrain dopamine signalling, through increased expression of nucleus accumbens dopamine D2 receptors, reduced expression of midbrain dopamine D2 autoreceptors or chemogenetic mesocorticolimbic dopamine neuron activation, increased responding under a PR schedule (Boekhoudt et al. 2018; Boender et al. 2014; de Jong et al. 2015; Trifilieff et al, 2013). Recently, behavioural economic studies have yielded comparable results for the role of dopamine in demand elasticity. Blockade of dopaminergic neurotransmission, using the dopamine receptor antagonist haloperidol, the dopamine depleting agent tetrabenazine, or genetic deletion of dopamine D2 receptors, led to increased elasticity of demand (Salamone et al. 2017; Soto et al. 2016). Conversely, demand elasticity for cocaine was found to decrease upon chemogenetic midbrain dopamine neuron activation (Mahler et al. 2019). However, the way in which mesocorticolimbic dopamine signalling is involved in cost–benefit trade-offs remains incompletely understood, as this may vary depending on the type of costs (i.e. physical or cognitive effort), for example. A full characterisation of the relationship between price and consumption may provide insight into how dopamine modulates the willingness to work for a reward as the effort-based costs change.

Therefore, the purpose of this study was to determine the role of dopaminergic neurotransmission in the relationship between price and consumption of sucrose, using a chemogenetic approach in rats. We selectively activated dopamine neurons in the VTA and determined the effects on demand elasticity and demand intensity for sucrose in a within-session approach. Motivation for sucrose was also assessed in the same animals under a PR schedule of reinforcement. To further gauge the role of dopamine in cost–benefit trade-offs, the effects of treatment with D-amphetamine and the dopamine receptor antagonist alpha-flupentixol were assessed. We hypothesised that, in accordance with previous rodent studies, increased dopamine neurotransmission would increase responding for sucrose at higher costs, resulting in a decreased demand elasticity.

## Materials and methods

### Animals

A total of 49 male rats, comprising three experimental groups, were used in this study. Tyrosine hydroxylase (TH)::Cre transgenic rats (Witten et al. 2011) were bred in-house, by crossing heterozygous TH::Cre ± (cre +) rats with wild-type Long Evans mates. Experimental group I consisted of 17 TH::cre + rats. Experimental group II was a control group that consisted of 16 homozygous TH::Cre-/- (cre-) littermates of the TH::cre + rats. Experiments with group I and II were performed in two batches (batch 1: *n* = 13 of which 7 TH::cre + ; batch 2: *n* = 20 of which 10 TH::cre +) for practical reasons. Rats from experimental group I and II were approximately 10 weeks old and weighed 220–300 g at the time of surgery (bodyweight (g), mean ± SEM: batch 1, 280 ± 7; batch 2, 265 ± 4). Experimental group III consisted of 16 adult Lister Hooded rats (Charles River, Sulzfeld, Germany), weighing 200–250 g (approximately 8–10 weeks old) at the start of the experiment. Upon arrival, rats from experimental group III had 8 days for acclimatisation, after which operant training commenced. All animals were experimentally naive.

The rats were individually housed in Macrolon type III sawdust bedded cages (42.5 × 26.6 × 18.5 cm) with ad libitum access to tap water. Initially, all rats had ad libitum access to standard chow (Rat and Mouse Breeder and Grower Expanded-CRM(E), Special Diet Service, UK). After the completion of training under a fixed ratio (FR) 1 schedule of reinforcement (see below), rats were food restricted (4 g of normal chow per 100 g body weight on training and test days, 6 g per 100 g body weight on remaining days) to approximately 85% of their free-feeding body weight. A polycarbonate rat tunnel (9 × 9 × 15 cm), a wood block, and a tissue were provided for cage enrichment. The rats were kept under controlled temperature and humidity conditions (21 ± 2 °C and 50–70% humidity) on a reversed 12-h/12-h light/dark cycle (lights off at 7.00 AM to lights on at 7.00 PM) to allow for behavioural testing in the dark phase. Background noise was provided by a constantly playing radio. The rats were weighed and handled at least once per week throughout the course of the experiment. All experimental procedures were approved by the Animal Ethics Committee of Utrecht University and the Dutch Central Animal Testing Committee and were conducted in accordance with Dutch (Wet op de Dierproeven, 1996; Herziene Wet op de Dierproeven, 2014) and European legislation (Guideline 86/609/EEC; Directive 2010/63/EU).

### Surgery

**Fig. 1:**
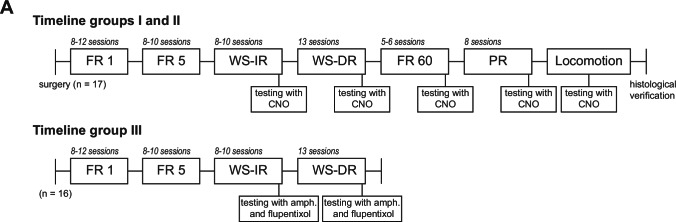
Schematic of experimental design. Timelines for experimental groups I, II and III. The number of training sessions before testing is indicated in italics above each block. WS-IR, within session increasing ratio; WS-DR, within session decreasing ratio; amph., D-amphetamine

Prior to behavioural training, the rats from experimental groups I and II underwent intracranial surgery. The rats were anaesthetised with a ketamine/dexmedetomidine mixture (0.2 ml mixture/100 g body weight, intraperitoneal: 75 mg/kg ketamine hydrochloride, Narketan, 0.25 mg/kg Dexdomitor®, Pfizer Animal Health B.V., the Netherlands). Local anaesthesia was provided by xylocaine, sprayed on the skull (Lidocaine 100 mg/ml, AstraZeneca BV), once the animals were placed in a stereotactic apparatus (Kopf Instruments). The rats were injected bilaterally into the VTA with 0.8 μl of AAV-hSyn-DIO-hM3Dq-mCherry (1.3 × 10^12^ genomic copies/ml; UNC Vector Core), using the following coordinates: AP − 5.5, ML + 1.3, DV − 8.1 (5° angle), in mm relative to Bregma. The virus was infused at a rate of 0.2 μl/min for 4 min, and the needle was left in place for 10 more min to allow for diffusion. Upon completion of the surgery, anaesthesia was terminated through the application of atipamezole (1.0 mg/kg, subcutaneously, Antisedan®, Pfizer Animal Health B.V., the Netherlands), and carprofen (5.0 mg/kg, subcutaneously, Carporal, AST Farma BV) was administered to the rats for pain relief on the day of surgery and the 2 following days. The rats were housed individually after surgery for the remainder of the experiment. Rats were housed 1 week under DM-II conditions after surgery to recover, followed by transportation to the animal facility where behavioural training and testing took place. After transportation, the rats had a minimum of eight days for acclimatisation to the reversed day light/dark cycle, after which operant training commenced (see Fig. 1 for the experimental outline).

### Drugs

Experimental groups I and II were treated with the selective DREADD ligand clozapine-N-oxide (CNO) or vehicle. CNO (Enzo Life Sciences BVBA, Belgium) was dissolved in milliQ and dissolved CNO was kept at 4 ˚C in between injections, for a maximum of 1 week. The doses of 0.3 mg/kg and 1.0 mg/kg CNO were chosen based on previous work (Boekhoudt et al. 2016, 2017b, 2018; Boender et al. 2014).

Experimental group III was treated with D-amphetamine and flupentixol. D-Amphetamine (d-amphetamine sulphate; Spruyt Hillen bv, the Netherlands) and flupentixol (cis-[Z]-α-flupentixol dihydrochloride; Sigma-Aldrich, the Netherlands) were dissolved in sterile saline (0.9% NaCl). Doses of 0.5 mg/kg and 1.0 mg/kg D-amphetamine and of 0.25 mg/kg and 0.5 mg/kg flupentixol were chosen based on previous work (Baarendse and Vanderschuren 2012; Baarendse et al. 2013; Boekhoudt et al. 2017a; Cardinal et al, 2000; Mayorga et al. 2000; Veeneman et al. 2011, 2012).

Prior to test injections, the rats were injected once with saline to habituate them to the injection procedure. All injections were given intraperitoneally at a volume of 1 ml per kg bodyweight. After injection, the rats were returned to their home cage, after which testing under the relevant training schedule started 30 min later. For all experiments, each rat received drug and vehicle injections according to a within-subjects Latin-square design.

### Apparatus

The rats were trained and tested in operant conditioning chambers (29.5 × 24 × 25 cm; Med Associates Inc., USA) equipped with two retractable levers (4.8 × 1.9 cm; ENV-112CM) and a white cue light (28 V, 100 mA; ENV-221 M) present above each lever. A recessed liquid dipper and food receptacle were situated in between the levers, equipped with an infrared beam for nose-poke detection. The wall on the opposite side of the box contained a white house light (28 V, 100 mA; ENV-215 M). The floor of the chamber was covered with a metal grid with bars separated by 1.57 cm. All chambers were situated in light- and sound-attenuating cubicles equipped with a ventilation fan and were controlled by MED-PC IV software (version 4.2) for Windows.

### Fixed ratio schedule of reinforcement

All rats were trained to respond for sucrose in 30-min operant sessions, once daily, 4–5 days per week. The house light was illuminated throughout the session. The position of the active and inactive levers was counterbalanced between rats. The animals were first trained to respond for sucrose under a fixed ratio (FR) 1 schedule of reinforcement. Pressing the active lever activated a pellet dispenser that delivered a 45 mg sucrose pellet (TestDiet, USA) into the food receptacle. Simultaneous with reward delivery, both levers were retracted, and the cue light above the active lever was illuminated until 1 s after the animal entered the food receptacle. Next, the cue light was turned off, and the levers were reintroduced, signalling the start of a new trial. All inactive lever presses were recorded but were without programmed consequences. After 8–12 FR 1 sessions, the animals were trained under a FR 5 schedule of reinforcement for sucrose for 8–10 sessions (Fig. 1).

### Within-session increasing/decreasing ratio schedule of reinforcement

Next, in order to determine demand curves, the rats were subjected to tests in which the ratio requirement increased within sessions (WS-IR) from 5 to 15, 30, 60 and 80. Each ratio requirement was offered for a block of 8 min separated by a 2-min inter-block interval that was signalled by retraction of the levers. Subsequently, the animals were trained in sessions in which the ratio requirement decreased within sessions (WS-DR), from 80 to 60, 30, 15 and 5. Similar to WS-IR sessions, each session consisted of five blocks of 8 min, separated by 2-min inter-block intervals. Experimental groups I, II and III were tested in WS-IR sessions, after 8–10 training sessions, and in WS-DR, after 13 training sessions (Fig. 1).

### Fixed ratio 60 schedule of reinforcement

After completion of the WS-IR and WS-DR tests, experimental groups I and II were trained (5–6 sessions) and tested under a FR 60 schedule of reinforcement (Fig. 1). This schedule had a similar block design as the WS-IR and WS-DR ratio sessions. In an FR 60 session, 60 active lever presses were required to obtain a sucrose pellet in all five blocks of 8 min each, with 2-min inter-block intervals in between.

### Progressive ratio schedule of reinforcement

Experimental groups I and II were subsequently trained (for 8 sessions) and tested under a progressive ratio (PR) schedule of reinforcement (Fig. 1), in which the response requirement for a sucrose pellet progressively increased after each obtained reward (response requirement: 1, 2, 4, 6, 9, 12, 15, 20, 25, etc.; Richardson and Roberts 1996). A PR session ended when no reward was earned for 30 consecutive min.

### Locomotor activity

Locomotor activity was assessed in experimental groups I and II (Fig. 1). Subjects were placed individually in smooth, grey-painted plastic arenas (50L × 30 W × 40H cm) 30 min after injection. Horizontal locomotor activity was registered using a camera positioned approximately 2 m above the setup. Distance travelled (cm) and velocity (cm/s) were recorded and analysed using video tracking software (EthoVision XT 13, Noldus, Wageningen, the Netherlands) which determined the position of the animals five times per second. Locomotor activity was measured for 1 h.

### Tissue preparation and immunohistochemical analysis

The animals from experimental groups I and II were euthanised by an intraperitoneal injection of sodium pentobarbital (0.2 ml/100 g; Euthanimal 1,709,296–08, Alfasan (Woerden, the Netherlands)), followed by a transcardial perfusion with 1 × phosphate buffered saline (PBS) followed by 4% paraformaldehyde (PFA) (P6148, Sigma-Aldrich (Zwijndrecht, the Netherlands)) in PBS. Brains were dissected and post-fixed in 4% PFA in PBS at 4 ˚C for at least 24 h, after which they were transferred to 30% sucrose in PBS at 4 °C for at least 3 days.

Using a cryostat, 40 μm coronal slices were cut and stored in PBS with 0.05% sodium azide. The slices were washed 3 × 15 min in PBS and then blocked for 1 h in PBS containing 10% v/v normal goat serum (Ab156046, Abcam plc, UK) and 0.25% v/v Triton-X100 at room temperature. Next, the slices were placed in PBS containing the primary antibodies (Rabbit anti-dsRed 1:750, #632,496, Clontech, Takara Bio USA Inc., USA; Mouse anti-Th 1:750, MAB318, Sigma-Aldrich) and 10% normal goat serum overnight at 4 °C. At room temperature, the slices were subsequently washed 3 × 15 min in PBS and placed in PBS containing the secondary antibodies (Goat anti-Rabbit 568, 1:750, A11011, Thermo Fisher; Goat anti-Mouse 488, 1:750, ab150113, Abcam plc, UK) and 2% normal goat serum for 2 h in the dark. Finally, the slices were washed 3 × 15 min in PBS and mounted onto microscope slides (Thermo Superfrost), dried and covered using Fluorsave (EMD Millipore Corporation, USA) and a coverslip.

To check for co-localisation of TH and hM3Dq-mCherry expression, images were captured at 2 × magnification using Olympus BX60 upright microscope and Leica Application Suite software (Leica Microscopy B.V., the Netherlands). Slides were illuminated with bright-field, fluorescein isothiocyanate (FITC) (515 nm; green) and tetramethylrhodamine (TRITC) (640 nm; red).

### Exclusion criteria

Two animals, both TH::cre + , from batch 1, were excluded from the experiment before operant training started because they did not recover sufficiently from surgery. Histological verification of infusion sites and viral expression was performed as an inclusion criterion. For experimental group I (i.e. TH::cre + rats), only animals that showed bilateral expression of hM3Dq-mCherry in the VTA were included in analyses. Two TH::cre + rats were excluded because no viral expression was detected. For experimental group II (i.e. TH::cre- rats), all animals were included in analyses as none of them showed expression of hM3DGq-mCherry.

### Data analysis

The number of rewards obtained during the WS-IR, WS-DR, FR 60 and PR sessions was measured per subject. Sessions in which an animal obtained < 5 rewards were excluded from further analyses since such low levels of responding hamper reliable analyses of the data. Based on this criterion, we had to exclude six animals from the flupentixol analyses as their response rates were strongly diminished when tested with the 0.5 mg/kg flupentixol dose. To avoid bias, all data from the 0.5 mg/kg flupentixol dose was therefore excluded from further analyses. Moreover, one animal from experimental group II was excluded from analysis of the WS-DR data, and one animal from experimental group III was excluded from analysis of the flupentixol treatment during WS-DR data due to obtaining < 5 rewards.

For experimental groups I and II, the effects of CNO treatment on the number of rewards obtained under the WS-IR, WS-DR and FR60 schedules were analysed using repeated measures analysis of variance (ANOVA) tests with dose and block as within-subject variables and group (TH::cre + or TH::cre-) as the between-subject variable. Number of rewards and breakpoint data from PR sessions were analysed using repeated measures ANOVAs with dose as the within-subject variable and group (TH::cre + or TH::cre-) as the between-subject variable. Since behavioural effects of CNO treatment compared to vehicle did not differ between experimental batches, these data were pooled for analysis. For experimental group III, the effects of D-amphetamine and flupentixol on the number of rewards were analysed using repeated measures ANOVAs with dose and block as the within-subject variables.

Subsequently, a behavioural demand analysis was executed on the data (i.e. number of rewards obtained) derived from the WS-IR and WS-DR sessions. Demand can be modelled using the exponential demand function (Hursh and Silberberg 2008). The exponential demand function is defined as: $\mathrm{log}Q=\mathrm{log}({Q}_{0})+k \times ({e}^{-\alpha \times {Q}_{0} \times C}-1)$. In this function, $Q$ is units of consumption (i.e. 3 rewards equals Q = 3), ${Q}_{0}$ is consumption at a minimally constrained price, and $C$ is the cost requirement (i.e. FR 15 would have $C$ = 15). Parameter $k$ is the number of logarithmic units spanned by the demand curve *e* indicates the base of the natural logarithm and $\alpha$ represents the rate constant of the exponential. Both $\alpha$ and ${Q}_{0}$ are estimated from the best fit function. Additionally, the gauge of the substantive significance of the model is given as *R*^2^. The *R*^2^ reflects how well the model fits the data by denoting the proportion of data variance that the equation accommodates.

Consumption during the WS-IR and WS-DR sessions was measured per subject and per ratio with units defined as ‘number of rewards earned’. To prevent zero values and to permit logarithmic transformations, 0.001 was added to each consumption value. Next, exponential demand functions, as detailed above, were fit to the data using the GraphPad Prism template kindly provided by the Institute for Behavioral Resources, Inc. website (http://ibrinc.org/software/). The overall mean performance was first analysed to determine the best-fitting $k$ parameter, which was used across all demand curve fits. For experimental groups I and II, the value of the $k$ parameter used was 2.116, and for experimental group III, the best-fitting $k$ parameter used was 1.667. Separate demand curves were fit to consumption values for individual subjects to determine individually fit $\alpha$ and ${Q}_{0}$ values and *R*^2^. Curves, and derived $\alpha$ and ${Q}_{0}$ values, with poor model fit (*R*^2^ ≤ 0.30), were discarded from analyses (Bentzley et al. 2013; Cohn et al. 2020; Fragale et al. 2017; Leonard et al. 2017; Murphy et al. 2009). Due to poor model fit, a total of five individual curves was excluded: one curve from the WS-DR in experimental group I, three curves from the WS-IR in experimental group II and three curves from the WS-DR in experimental group III.

Behavioural economics provides a framework for studying demand from the level of the individual through that of large numbers of consumers (Hursh & Roma, 2016; Hursh & Silberberg, 2008). Analysis of demand curves on a population level is common in economics to provide insights into a particular market. In addition, analysis of individual demand curves is also frequently used in a preclinical setting. To fully exploit the asset of the behavioural economics framework, demand curves were analysed both at an individual level as well as at a population level. Individually fit $\alpha$ and ${Q}_{0}$ values were used for the primary analysis to determine group and dose differences. For experimental groups I and II, ANOVAs were performed with dose as a within-subject variable and group (TH::cre + or TH::cre-) as the between-subject variable. For experimental group III, ANOVAs were performed with dose as a within-subject variable to assess the effects of D-amphetamine, and with paired *t* tests and Wilcoxon matched-pairs signed-rank tests to assess the effects of flupentixol. A secondary analysis on the demand functions per population was conducted using an extra sum-of-squares *F*-test to determine whether the best-fit values for demand curve parameters significantly differed over dosages. The null hypothesis was that parameters did not differ and therefore a single demand curve fit the data from different doses. A significant F-statistic indicated that a single demand curve could not accommodate data from different doses. In that case, separate demand curves per dose offer a better accommodation of the data.

Horizontal locomotor activity was expressed as travelled distance (cm) in 5 min time bins. The effects of CNO administration on locomotor activity were analysed in experimental groups I and II using a repeated measures ANOVA tests with dose and time bin as the within-subject variables and group (TH::cre + or TH::cre-) as the between-subject variable.

For every ANOVA and *t* test, normal distribution of the data was assessed. Locomotor activity, demand elasticity variables derived from experimental group I and II, and demand elasticity variables from D-amphetamine tests derived from experimental group III were transformed prior to statistical analyses by natural log and again checked for normal distribution prior to parametric testing. Whenever the difference scores of the flupentixol measurements compared to vehicle were not normally distributed, a Wilcoxon matched-pairs signed-rank test was used. This was the case for the demand elasticity WS-IR and demand intensity WS-DR variables. A total of twelve statistical outliers were removed from the analyses: three datapoints from the WS-IR in experimental group I, three datapoints from the WS-IR in experimental group II and six datapoints from experimental group III. Mauchly’s test of sphericity was used to test whether variances of the differences between levels were equal. If the assumption of sphericity was violated, degrees of freedom were corrected using Greenhouse–Geisser (GG) estimates of sphericity or Huynh–Feldt estimates of sphericity when the GG estimate was > 0.75. Corrected degrees of freedom are presented rounded to the nearest integer. When significant main effects or interactions were detected, post hoc analyses were conducted using pairwise comparisons with Bonferroni corrections.

Data were analysed and visualised using Microsoft Excel, GraphPad Prism (version 8.3.0, GraphPad Software Inc., USA) and SPSS for Windows (version 25.0.0.1, IBM Corp., USA). Results are presented as mean ± SEM unless otherwise stated. A significance criterion of *p* < 0.05, two-tailed, was used for all statistical analyses.

## Results

### Experimental group I and II: effects of chemogenetic activation of VTA dopamine neurons

#### Virus expression

**Fig. 2:**
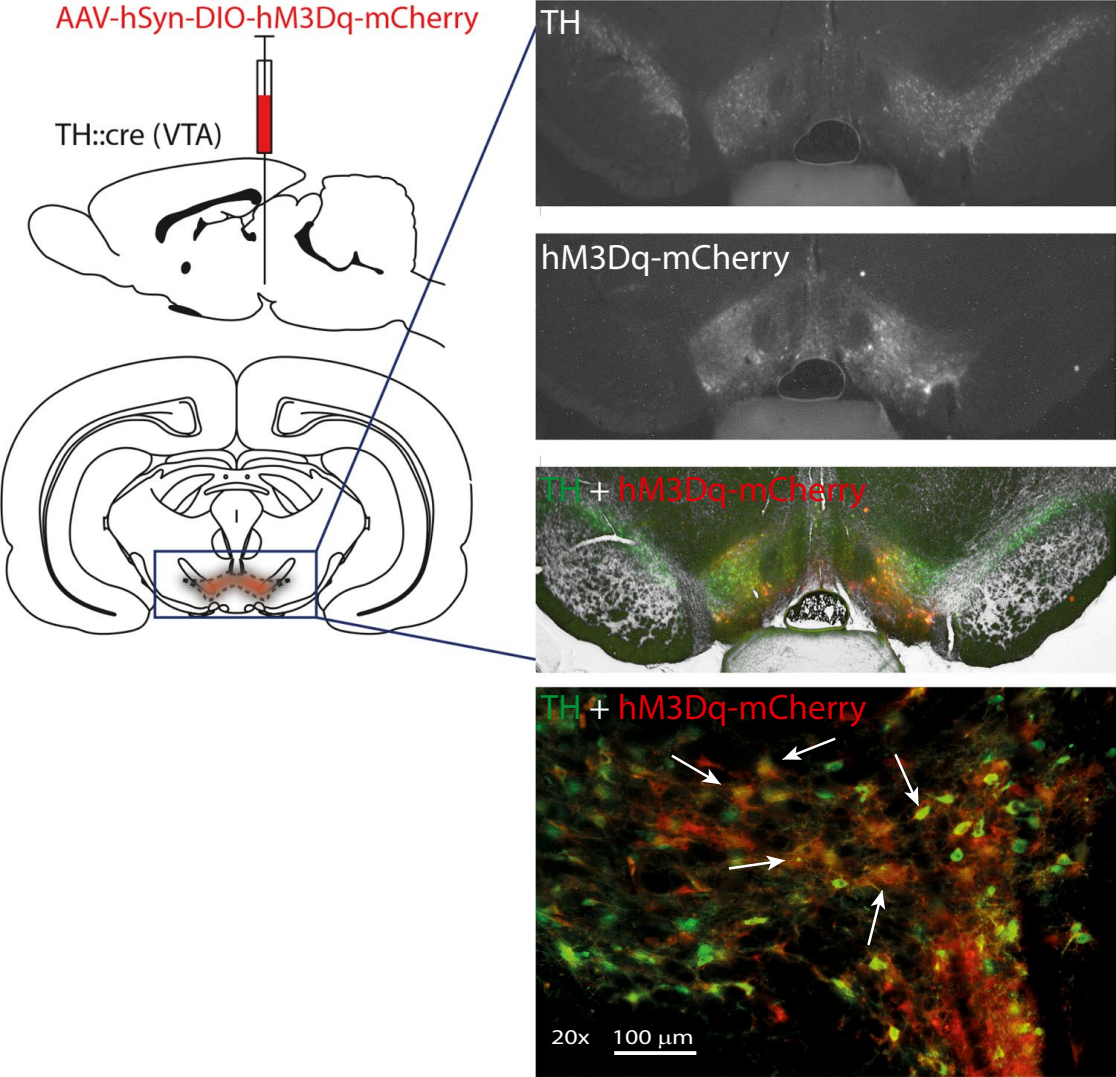
Expression of AAV-hSyn-DIO-hM3Dq-mCherry in experimental group I (TH::cre +) animals. Representative example of a TH::cre + rat injected with AAV-hSyn-DIO-hM3Dq-mCherry of the ventral tegmental area (VTA). Expression is shown in coronal slices − 5.6 mm posterior to Bregma. The arrows highlight examples of neurons that co-express TH and mCherry. Atlas image adapted from Paxinos and Watson, 2004

Immunohistochemical analysis confirmed DREADD expression (hM3Dq-mCherry) in dopamine neurons throughout the VTA in TH::cre + animals (Fig. 2). In absence of Cre (i.e., in TH::cre- animals), no DREADD expression was observed (data not shown).

## Within-session increasing ratio

**Fig. 3:**
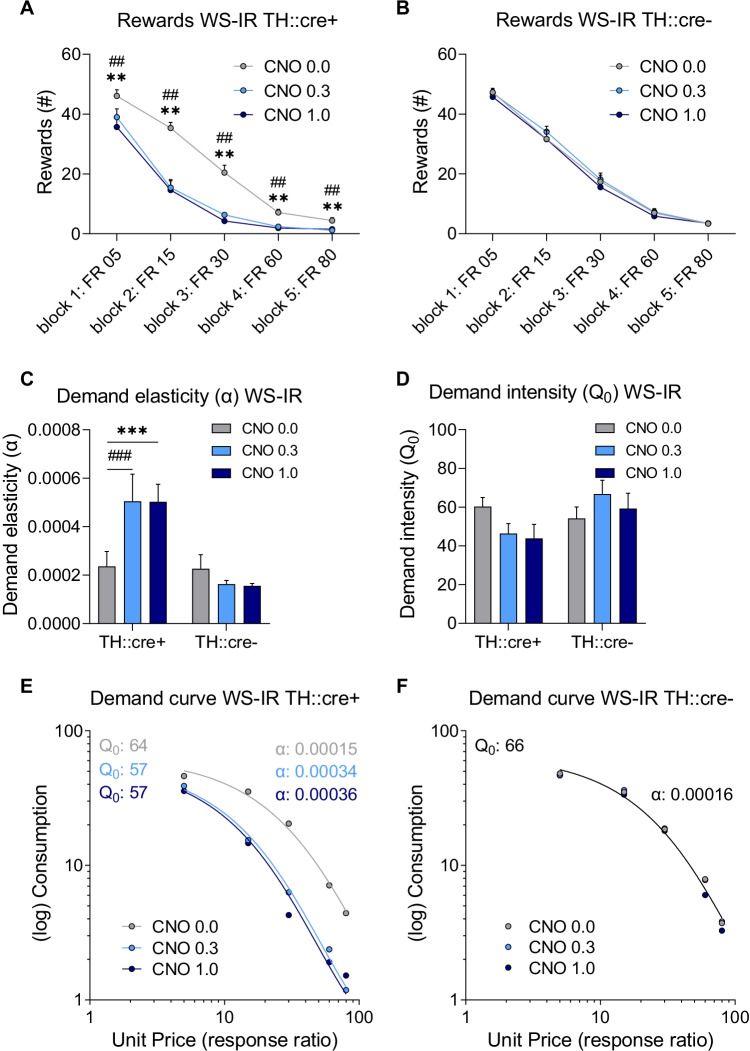
The effects of CNO treatment on the number of rewards, demand elasticity (*α*) and intensity (*Q*_*0*_), and demand curve when measured under a within session increasing ratio (WS-IR) schedule in experimental groups I (TH::cre +) and II (TH::cre-). Effects of CNO on number of rewards obtained in TH::cre + rats (**A**) and TH::cre- rats (**B**) when assessed in a WS-IR task. Effects of CNO on demand elasticity (**C**) and demand intensity (**D**) based on individual demand curve analysis. Effects of CNO on population demand curve in TH::cre + rats (**E**) and TH::cre- rats (**F**). Data in panels **A**–**D** are presented as the mean + SEM. ** CNO 1.0 different from vehicle, *p* < 0.01; ## CNO 0.3 different from vehicle, *p* < 0.01; *** CNO 1.0 different from vehicle, *p* < 0.001; ### CNO 0.3 different from vehicle, *p* < 0.001

Responding for sucrose was assessed following treatment with CNO under a within-session increasing ratio (WS-IR) schedule of reinforcement. CNO treatment significantly reduced the number of rewards obtained in TH::cre + , but not in TH::cre- rats, and this effect was dependent on block (Fig. 3A–B; F(2,40)_dose_ = 21.861, *p* < 0.001; F(2,40)_dose × group_ = 19.724, *p* < 0.001; F(4,116)_dose × block_ = 5.409, *p* < 0.001; F(4,116)_dose x block × group_ = 6.473, *p* < 0.001). Post hoc analyses showed that both the 0.3 mg/kg and 1.0 mg/kg CNO dose reduced the number of rewards obtained compared to vehicle in every block in TH::cre + animals (*p* < 0.01). In contrast, CNO treatment had no significant effect on the number of rewards in any of the blocks in TH::cre- animals. Thus, chemogenetic activation of VTA dopamine neurons decreased the number of rewards obtained across the ratio requirements.

Individual demand curves were plotted, and the derived parameters *R*^2^, $\alpha$, and ${Q}_{0}$ were compared. For all treatments, individual demand data fitted well to the model as the average *R*^2^ was above 0.80 (i.e. [mean ± standard deviation] *R*^2^_vehicle_: 0.85 ± 0.21; *R*^2^_CNO 0.3_: 0.89 ± 0.12; *R*^2^_CNO 1.0_: 0.86 ± 0.18). CNO treatment significantly increased demand elasticity in TH::cre + , but not in TH::cre- rats (Fig. 3C; F(2,42)_dose_ = 9.101, p = 0.001; F(2,42)_dose × group_ = 19.854, *p* < 0.001). Post hoc analyses showed that both the 0.3 mg/kg and the 1.0 mg/kg CNO dose increased demand elasticity compared to vehicle in TH::cre + animals (*p* < 0.001). In contrast, CNO treatment had no significant effect on demand elasticity in TH::cre- animals. Moreover, demand intensity (${Q}_{0}$) was not affected by CNO treatment in either group (Fig. 3D; F(2,42)_dose_ = 0.553, *p* = 0.579; F(1,21)_group_ = 2.096, *p* = 0.162; F(2,42)_dose × group_ = 2.925, *p* = 0.065). Taken together, chemogenetic activation of VTA dopamine neurons increased demand elasticity without significantly affecting demand intensity.

Next, demand curves based on group means were plotted and analysed separately per TH::cre group. The extra sum-of-squares *F*-test showed that a global fit could not accommodate all data in the TH::cre + group (F(4,9) = 28.000, *p* < 0.001). Therefore, demand curves and the derived best-fit values of $\alpha$ and ${Q}_{0}$ were different for each dose in the TH::cre + group, with an increased $\alpha$ and decreased ${Q}_{0}$ when under the influence of CNO (Fig. 3E). In the TH::cre- group, the extra sum-of-squares *F*-test indicated that parameters did not differ across the different doses and that a single demand curve with an *R*^2^ of 0.99 fit the data from different doses (Fig. 3F; F(4,9) = 0.930, *p* = 0.489). This confirms that CNO treatment had no significant effect on demand in the TH::cre- animals. Thus, analysis of group demand curves suggests that chemogenetic activation of VTA dopamine neurons shifted the demand curve to one with an increased demand elasticity and decreased demand intensity (${Q}_{0}$).

## Within-session decreasing ratio

To minimise potential satiety effects that might arise as the session and number of obtained rewards progressed, the animals were also tested under a reversed schedule of reinforcement (i.e. within-session decreasing ratio, WS-DR). Similar ratio requirements as for the WS-IR schedule of reinforcement were used, but the ratio requirements were presented in a reversed order, such that the required effort per reward decreased over blocks.

**Fig. 4:**
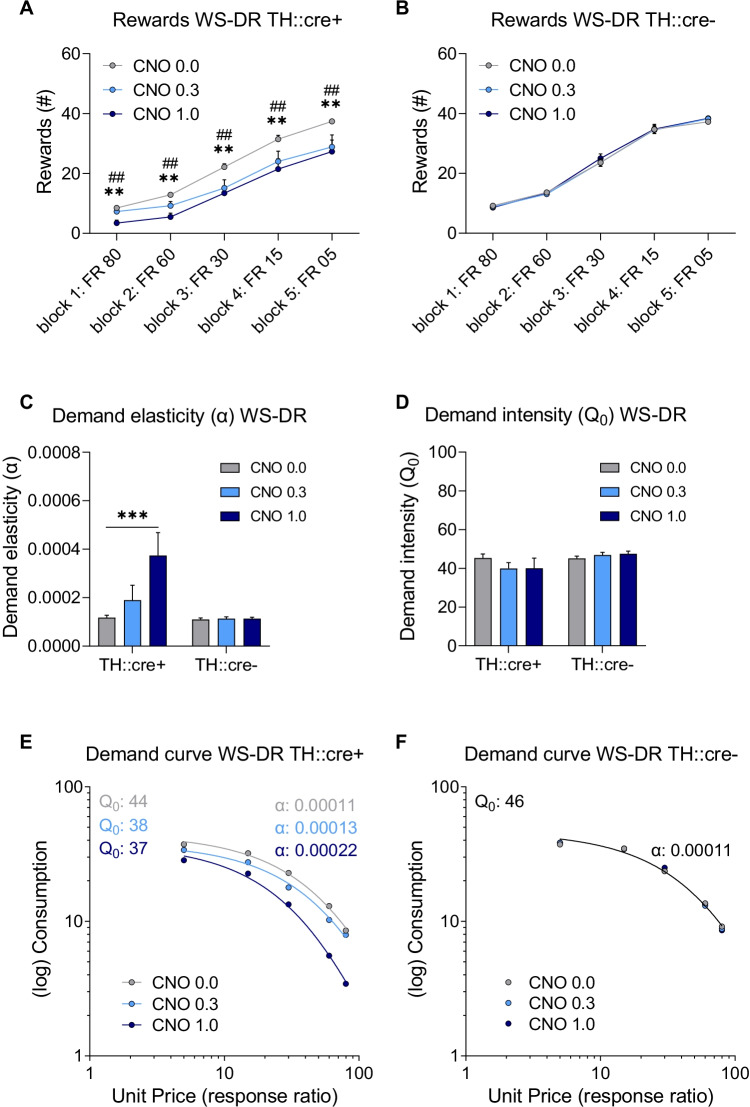
The effects of CNO treatment on the number of rewards, demand elasticity (*α*) and intensity (*Q*_*0*_) and demand curve when measured under a within session decreasing ratio (WS-DR) schedule in experimental groups I (TH::cre +) and II (TH::cre-). Effects of CNO on number of rewards obtained in TH::cre + rats (**A**) and TH::cre- rats (**B**) when assessed in a WS-DR task. Effects of CNO on demand elasticity (**C**) and demand intensity (**D**) based on individual demand curve analysis. Effects of CNO on population demand curve in TH::cre + rats (**E**) and TH::cre- rats (**F**). Data in panels **A**–**D** are presented as the mean + SEM. ** CNO 1.0 different from vehicle, *p* < 0.01; ## CNO 0.3 different from vehicle, *p* < 0.01; *** CNO 1.0 different from vehicle, *p* < 0.001

Analysis of the data for responding under the WS-DR schedule of reinforcement revealed that CNO treatment significantly decreased the number of rewards obtained in TH::cre + , but not in TH::cre- rats, and the effect was not dependent on block (Fig. 4A–B; F(2,52)_dose_ = 7.366, *p* = 0.002; F(2,52)_dose × group_ = 8.606, *p* = 0.001; F(4,116)_dose × block_ = 1.043, *p* = 0.392). Post hoc analyses showed that both the 0.3 mg/kg and 1.0 mg/kg CNO dose decreased the number of earned rewards compared to vehicle in TH::cre + animals (*p* < 0.005). This effect of CNO was consistent over blocks (F(4,116)_dose × block × group_ = 1.813, *p* = 0.124). In contrast, CNO treatment had no significant effect on the number of rewards in TH::cre- animals. Thus, chemogenetic activation of VTA dopamine neurons decreased the number of rewards earned across the ratio requirements.

Individual demand curves were plotted, and the derived parameters *R*^2^, $\alpha$, and ${Q}_{0}$ were compared. For all treatments, individual demand data fitted well to the model as the average *R*^2^ was above 0.90 (i.e. [mean ± standard deviation] *R*^2^_vehicle_: 0.96 ± 0.04; *R*^2^_CNO 0.3_: 0.95 ± 0.10; *R*^2^_CNO 1.0_: 0.93 ± 0.08). CNO treatment significantly increased demand elasticity in TH::cre + , but not in TH::cre- rats (Fig. 4C; F(2,48)_dose_ = 11.623, *p* < 0.001; F(2,48)_dose × group_ = 10.552, *p* < 0.001). Post hoc analyses showed that the 1.0 mg/kg CNO dose increased demand elasticity compared to vehicle in TH::cre + animals (*p* < 0.001). In contrast, CNO treatment had no effect on demand elasticity in TH::cre- animals. Moreover, demand intensity (${Q}_{0}$) was not affected by CNO treatment in either group (Fig. 4D; F(1,24)_group_ = 3.211, *p* = 0.086; F(1,33)_dose_ = 0.483, *p* = 0.551; F(1,33)_dose × group_ = 2.286, *p* = 0.132). These findings indicate that chemogenetic activation of VTA dopamine neurons increased demand elasticity without significantly affecting demand intensity.

Next, demand curves based on group means were plotted and analysed separately per TH::cre group. The extra sum-of-squares *F*-test showed that a global fit could not accommodate all data in the TH::cre + group (F(4,9) = 86.000, *p* < 0.001). Demand curves and the derived best-fit values of $\alpha$ and ${Q}_{0}$ were different for each dose in the TH::cre + group, with an increased $\alpha$ and decreased ${Q}_{0}$ when under the influence of CNO (Fig. 4E). In the TH::cre- group, the extra sum-of-squares *F*-test indicated that parameters did not differ across the different doses and that a single demand curve with an *R*^2^ of 0.99 fit the data from different doses (Fig. 4F; F(4,9) = 0.160, *p* = 0.952). Together, these group demand curve analyses revealed that chemogenetic activation of VTA dopamine neurons shifted the demand curve to one with an increased demand elasticity and decreased demand intensity (${Q}_{0}$).

## Fixed ratio 60

As these results were obtained in sessions in which the required effort per reward changed over time blocks, a certain level of behavioural flexibility might be required, which may be compromised as a result of hyperactivity of VTA dopamine cells (Floresco 2013; Izquierdo et al. 2017; Verharen et al. 2018, 2019). Therefore, to control for potential CNO treatment effects on flexibility in reward seeking behaviour, responding of the animals, following treatment with CNO or vehicle, was also assessed under a FR 60 schedule of reinforcement. In this schedule, the required effort per reward was high but did not change over time blocks.

**Fig. 5:**
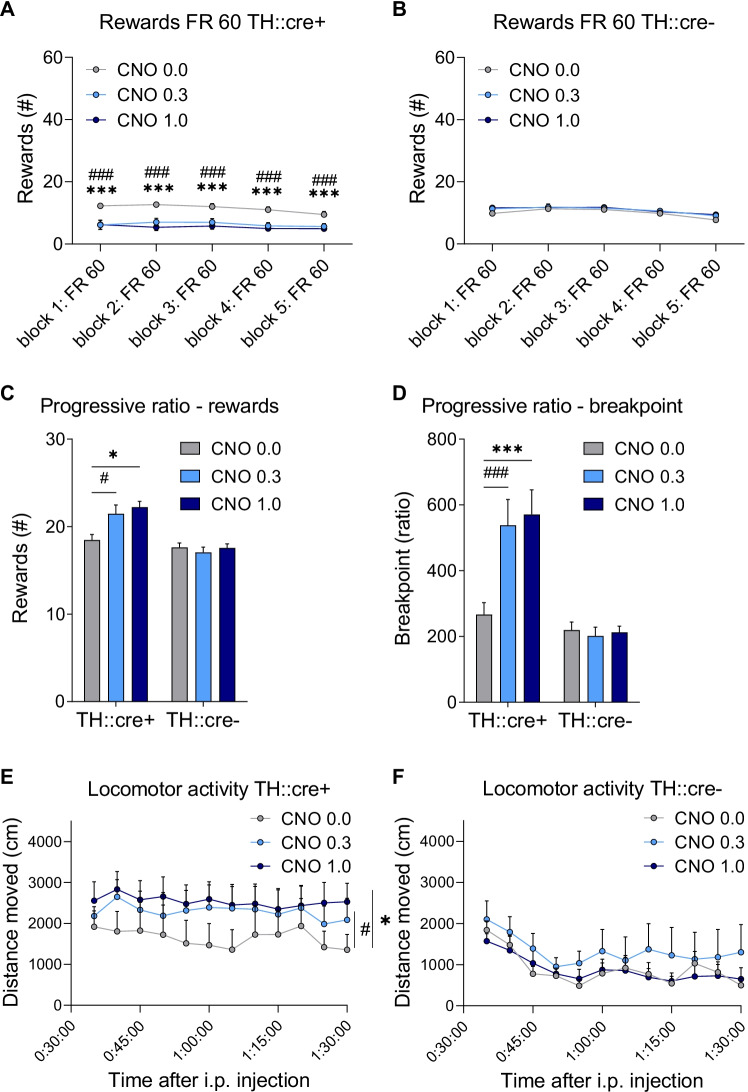
The effects of CNO treatment on performance in fixed ratio (FR) 60 sessions, progressive ratio (PR) sessions and locomotor activity in experimental groups I (TH::cre +) and II (TH::cre-). Effects of CNO on number of rewards obtained in TH::cre + rats (**A**) and TH::cre- rats (**B**) when assessed in a FR 60 task. Effects of CNO on number of rewards obtained (**C**) and breakpoint (**D**) in TH::cre + and TH::cre- rats when assessed in a PR task. Effects of CNO on distance moved in TH::cre + rats (**E**) and TH::cre- rats (**F**) when locomotor activity was assessed. Data are presented as the mean + SEM. * CNO 1.0 different from vehicle, *p* < 0.05; # CNO 0.3 different from vehicle, *p* < 0.05; *** CNO 1.0 different from vehicle, *p* < 0.001; ### CNO 0.3 different from vehicle, *p* < 0.001

CNO treatment significantly decreased the number of rewards obtained in TH::cre + , but not in TH::cre- rats, and the effect was not dependent on block (Fig. 5A–B; F(1,39)_dose_ = 13.266, *p* < 0.001; F(1,39)_dose × group_ = 27.009, *p* < 0.001; F(5,137)_dose × block_ = 1.623, *p* = 0.157). Post hoc analyses showed that both the 0.3 mg/kg and the 1.0 mg/kg CNO dose decreased the number of rewards earned compared to vehicle in TH::cre + animals (*p* < 0.001). This effect of CNO in the TH::cre + group was consistent over blocks (F(5,137)_dose × block × group_ = 0.650, *p* = 0.664). In contrast, CNO treatment had no effect on the number of rewards in the TH::cre- animals. The number of rewards obtained decreased during the session as an effect of block was observed, but this was not different between the groups (F(2,63)_block_ = 12.136, *p* < 0.001; F(2,63)_block × group_ = 0.760, *p* = 0.491). Post hoc analyses showed that the number of rewards earned was significantly lower in the final block compared to all other blocks (*p* < 0.05), which might reflect a satiation effect. Thus, chemogenetic activation of VTA dopamine neurons decreased the number of obtained rewards even when flexibility was not required in the task.

## Progressive ratio

Besides demand curve analyses, the effects of CNO treatment on motivation were assessed using a PR schedule of reinforcement in the same animals. Dopamine has been strongly implicated in incentive motivation (Salamone and Correa, 2012), and incentive motivational aspects can influence the cost–benefit trade-offs. Therefore, assessment of the effects of CNO treatment on PR performance would provide a more detailed understanding of the role of dopaminergic neurotransmission in incentive motivational aspects and the relationship between price and consumption of sucrose.

CNO treatment significantly increased the number of rewards obtained and the breakpoint in TH::cre + , but not in TH::cre- rats (Fig. 5C–D; rewards: F(2,46)_dose_ = 7.153, *p* = 0.003; F(2,46)_dose × group_ = 9.343, *p* = 0.001; breakpoint: F(2,54)_dose_ = 10.847, *p* < 0.001; F(2,54)_dose × group_ = 12.715, *p* < 0.001). Post hoc analyses showed that both the 0.3 mg/kg and 1.0 mg/kg CNO dose increased the number of rewards and the breakpoint compared to vehicle in TH::cre + animals (rewards: *p* < 0.05; breakpoint: *p* < 0.001). In contrast, CNO treatment had no effect on the number of rewards and the breakpoint in TH::cre- animals. These results show that chemogenetic activation of VTA dopamine neurons increased responding for sucrose under a PR schedule of reinforcement.

## Locomotor activity

The effect of CNO on locomotor activity, which is not directly related to food motivation, was assessed as a functional control for activation of VTA dopamine neurons. Chemogenetic activation of these neurons was previously shown to induce a hyperactive phenotype (Boekhoudt et al. 2016; Wang et al. 2013). CNO treatment significantly increased the distance travelled in TH::cre + , but not in TH::cre- rats (Fig. 5E–F; F(2,46)_dose_ = 4.741, *p* = 0.018; F(2,46)_dose × group_ = 3.370, *p* = 0.049). Post hoc analyses showed that both the 0.3 mg/kg and 1.0 mg/kg CNO dose increased distance travelled compared to vehicle in TH::cre + animals (*p* < 0.05). By contrast, CNO treatment had no effect on locomotor activity in TH::cre- animals. The effect of CNO was constant throughout the test (F(7,200)_dose × time bin_ = 0.997, *p* = 0.437; F(7,200)_dose × time bin × group_ = 0.735, *p* = 0.651). Thus, chemogenetic activation of VTA dopamine neurons increased locomotor activity in TH::cre + rats.

### Experimental group III: effects of D-amphetamine and flupentixol

## Within-session increasing ratio

**Fig. 6:**
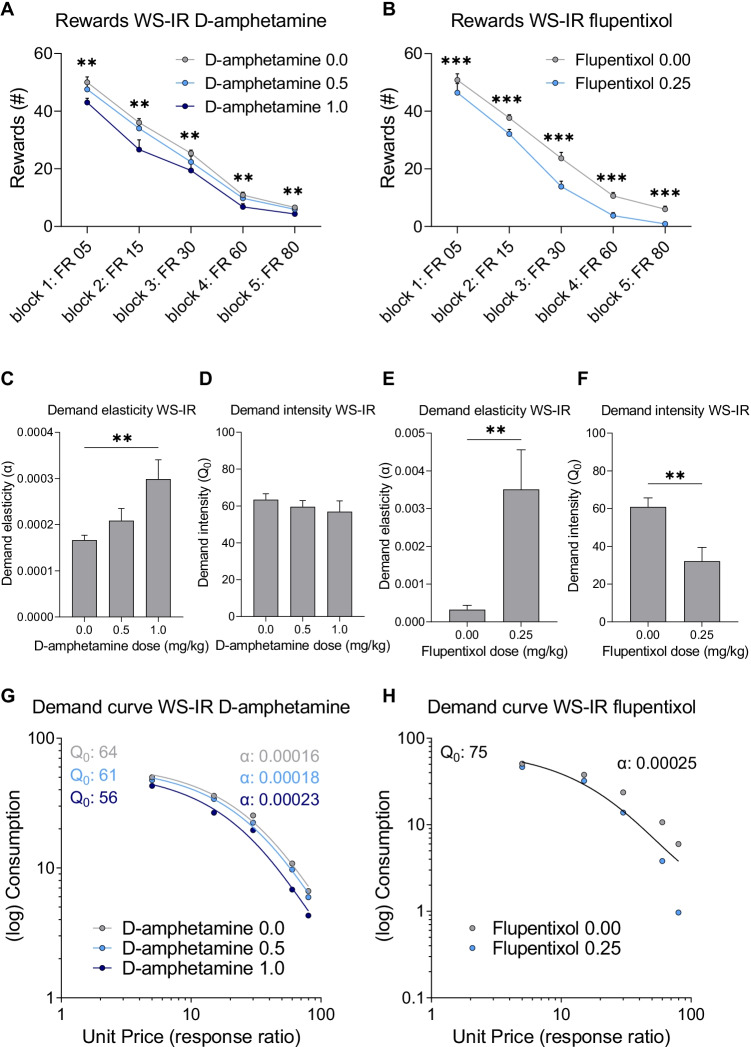
The effects of D-amphetamine and flupentixol treatment on the number of rewards, demand elasticity (*α*) and intensity (*Q*_*0*_)and demand curve when measured under a within session increasing ratio (WS-IR) schedule in experimental group III. Effects of D-amphetamine (**A**) and flupentixol (**B**) on number of rewards obtained when assessed in a WS-IR task. Effects of D-amphetamine on demand elasticity (**C**) and demand intensity (**D**) based on individual demand curve analysis. Effects of flupentixol on demand elasticity (**E**) and demand intensity (**F**) based on individual demand curve analysis. Effects of D-amphetamine (**G**) and flupentixol (**H**) on population demand curves. Data in panels **A**–**D** are presented as the mean + SEM. ** different from vehicle, *p* < 0.01; *** different from vehicle, *p* < 0.001

Responding for sucrose under a WS-IR schedule of reinforcement was determined following systemic treatment with D-amphetamine and flupentixol. D-Amphetamine treatment significantly decreased the number of rewards obtained (Fig. 6A; F(1,21)_dose_ = 9.958, *p* = 0.002). Post hoc analysis showed that the number of rewards at the 1.0 mg/kg D-amphetamine dose was significantly lower when compared to vehicle treatment (*p* = 0.003). As the required ratio increased over blocks, the number of rewards obtained significantly decreased (F(3,38)_block_ = 319.872, *p* < 0.001). The dampening effect of D-amphetamine on the number of rewards obtained was independent of the ratio requirement (F(3,47)_dose × block_ = 1.224, *p* = 0.312). Flupentixol also significantly decreased the number of rewards obtained compared to vehicle (Fig. 6B; F(1,15)_dose_ = 34.013, *p* < 0.001). As the required ratio increased over blocks, the number of rewards obtained significantly decreased (F(2,27)_block_ = 242.688, *p* < 0.001). The dampening effect of flupentixol on number of rewards was not related to the ratio requirements (F(4,60)_dose × block_ = 2.038, *p* = 0.100). Taken together, these data show that both D-amphetamine and flupentixol reduced the number of rewards obtained under a WS-IR schedule of reinforcement.

Next, individual demand curves were plotted and the derived parameters *R*^2^, $\alpha$, and ${Q}_{0}$ were analysed for both D-amphetamine and flupentixol treatments. For D-amphetamine, individual demand data fitted well to the model as the average *R*^2^ was above 0.80 (i.e. [mean ± standard deviation] *R*^2^_D-amphetamine_: 0.93 ± 0.09; *R*^2^_D-amphetamine 0.5_: 0.88 ± 0.13; *R*^2^_D-amphetamine 1.0_: 0.80 ± 0.19). D-Amphetamine significantly increased demand elasticity (Fig. 6C; F(2,28)_dose_ = 8.493, *p* = 0.001). Post hoc analysis showed that demand elasticity at the 1.0 mg/kg D-amphetamine dose was significantly higher compared to vehicle (mean difference: 0.216, *p* = 0.001). D-Amphetamine did not significantly affect demand intensity (Fig. 6D; F(1,18)_dose_ = 1.232, *p* = 0.291). After flupentixol treatment, individual demand data fitted only marginally to the model as the average *R*^2^ values were relatively low (i.e. [mean ± standard deviation] *R*^2^_flupentixol 0.00_: 0.86 ± 0.19; *R*^2^_flupentixol 0.25_: 0.54 ± 0.23). Flupentixol significantly increased demand elasticity compared to vehicle (Fig. 6E; Z =  − 2.897, *p* = 0.004). Flupentixol also significantly reduced demand intensity (Fig. 6F; t(15) = 3.471, *p* = 0.003). Together, these results show that D-amphetamine increased demand elasticity without significantly affecting demand intensity, while flupentixol increased demand elasticity and decreased demand intensity.

Analysis of the demand curves based on dose means for D-amphetamine treatment using the extra sum-of-squares *F*-test indicated that a global fit could not accommodate all data (F(4,9) = 8.200, *p* = 0.005). Therefore, demand curves and the derived best-fit values of $\alpha$ and ${Q}_{0}$ were different for each D-amphetamine dose (Fig. 6G). These analyses suggest that D-amphetamine shifted the demand curve to one with an increased demand elasticity and decreased demand intensity.

For flupentixol treatment, the extra sum-of-squares *F*-test indicated that parameters did not differ across the different doses and that a single demand curve with an *R*^2^ of 0.80 fit the data from different doses (Fig. 6H; F(2,6) = 3.900, *p* = 0.082). This analysis suggests that flupentixol did not alter the demand curve.

## Within-session decreasing ratio

**Fig. 7:**
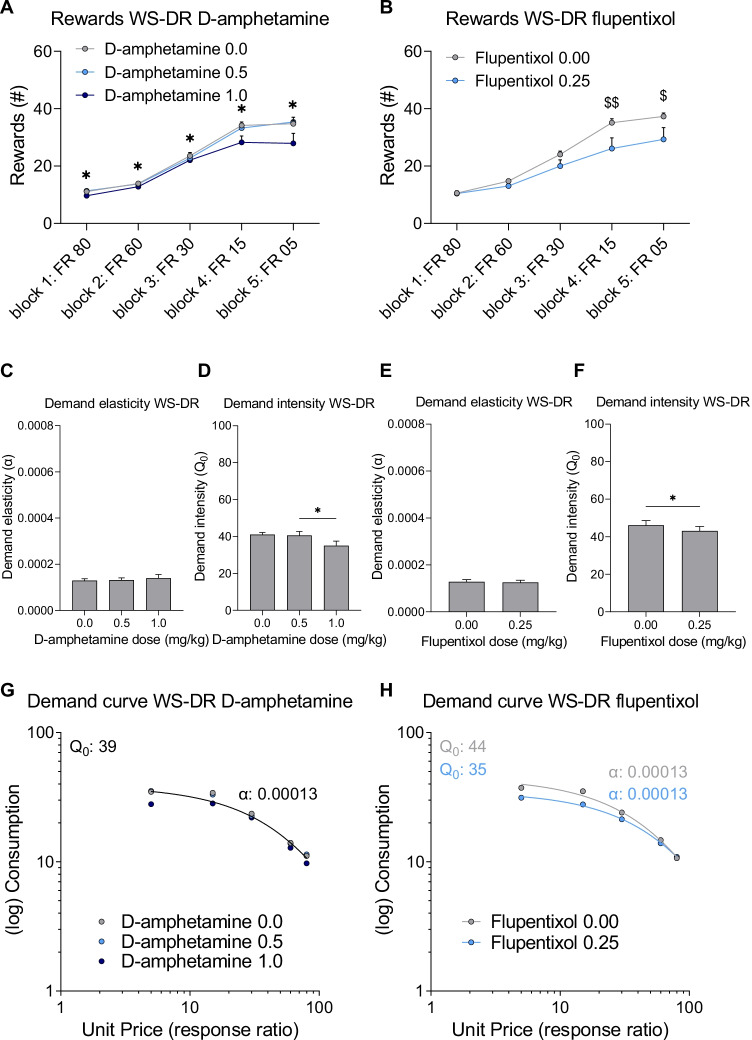
The effects of D-amphetamine and flupentixol treatment on the number of rewards, demand elasticity (*α*) and intensity (*Q*_*0*_) and demand curve when measured under a within session decreasing ratio (WS-DR) schedule in experimental group III. Effects of D-amphetamine (**A**) and flupentixol (**B**) on number of rewards obtained when assessed in a WS-DR task. Effects of D-amphetamine on demand elasticity (**C**) and demand intensity (**D**) based on individual demand curve analysis. Effects of flupentixol on demand elasticity (**E**) and demand intensity (**F**) based on individual demand curve analysis. Effects of D-amphetamine (**G**) and flupentixol (**H**) on population demand curves. Data in panels **A**–**D** are presented as the mean + SEM. (A) * D-amphetamine 1.0 mg/kg different from vehicle, *p* < 0.05; (B) $$ *p* = 0.053; $ *p* = 0.073 (D) * D-amphetamine 0.5 mg/kg different from D-amphetamine 1.0 mg/kg, *p* < 0.05; (F) * flupenthixol different from vehicle *p* < 0.05

The effects of D-amphetamine and flupentixol on responding for sucrose under a WS-DR schedule of reinforcement were also determined. D-Amphetamine treatment significantly decreased the number of rewards obtained (Fig. 7A; F(1,20)_dose_ = 6.421, *p* = 0.014). Post hoc analysis showed that the number of rewards at the 1.0 mg/kg D-amphetamine dose was significantly lower than vehicle (mean difference: 3.4, *p* = 0.038). As the required ratio decreased over blocks, the number of rewards obtained significantly increased (F(2,29)_block_ = 155.212, *p* < 0.001). The dampening effect of D-amphetamine on the number of rewards was not affected by the ratio requirement (F(2,35)_dose × block_ = 2.006, *p* = 0.144). Treatment with flupentixol did not significantly affect the number of rewards obtained, although a trend towards a decrease in the number of rewards was observed (Fig. 7B; F(1,14)_dose_ = 4.328, *p* = 0.056). As the required ratio decreased over blocks, the number of rewards obtained significantly increased (F(1,21)_block_ = 81.278, *p* < 0.001). The effect of flupentixol on the number of rewards was dependent on the ratio requirement (F(2,26)_dose × block_ = 3.531, *p* = 0.048). Post hoc analysis indicated no significant differences between flupentixol and vehicle treatment in any of the blocks, although a trend towards a suppression in responding by flupentixol was observed in block 4, i.e. FR 15 (*p* = 0.053), and in block 5, i.e. FR 5 (*p* = 0.073). Together, these results show that D-amphetamine significantly decreased the number of earned rewards and revealed a trend towards a reduction in the number of rewards upon treatment with flupentixol, especially at the lowest ratio requirements.

Individual demand curves were plotted and the derived parameters *R*^2^, $\alpha$, and ${Q}_{0}$ were analysed. For D-amphetamine treatment, individual demand data fitted well to the model as the average *R*^2^ was above 0.80 (i.e. [mean ± standard deviation] *R*^2^_D-amphetamine 0.0_: 0.94 ± 0.06; *R*^2^_D-amphetamine 0.5_: 0.92 ± 0.08; *R*^2^_D-amphetamine 1.0_: 0.83 ± 0.14). D-Amphetamine did not significantly affect demand elasticity (Fig. 7C; F(1,18) _dose_ = 0.049, *p* = 0.891). There was a significant effect of dose on demand intensity (Fig. 7D; F(2,28) _dose_ = 4.792, *p* = 0.016). Post hoc analyses showed that demand intensity at both D-amphetamine doses was not significantly different from vehicle (*p* > 0.07), but that both doses of D-amphetamine differed from each other. For flupentixol treatment, individual demand data fitted well to the model as the average *R*^2^ was above 0.90 (i.e. [mean ± standard deviation] *R*^2^_flupentixol 0.00_: 0.95 ± 0.03; *R*^2^_flupentixol 0.25_: 0.94 ± 0.02). Flupentixol did not significantly affect demand elasticity (Fig. 7E; t(11) = 0.433, *p* = 0.674). However, flupentixol significantly decreased demand intensity compared to vehicle (Fig. 7F; Z =  − 2.515, *p* = 0.012). Thus, treatment with D-amphetamine did not affect demand elasticity and demand intensity, whereas flupentixol decreased demand intensity without significantly affecting demand elasticity.

Analysis of the demand curves based on dose means for D-amphetamine treatment using the extra sum-of-squares *F*-test indicated that parameters did not differ across the different doses (F(4,9) = 2.600, *p* = 0.104). Therefore, a single demand curve with an *R*^2^ of 0.96 fit the data from different doses (Fig. 7G). These results suggest that D-amphetamine did not shift the demand curve. For flupentixol treatment, the extra sum-of-squares *F*-test showed that a global fit could not accommodate all data (F(2,6) = 12.000, *p* = 0.008). Therefore, demand curves and the derived best-fit value ${Q}_{0}$ were different for vehicle and flupentixol, with a similar $\alpha$ (Fig. 7H). Thus, analysis of mean demand curves suggested that flupentixol shifted the demand curve to one with a similar demand elasticity and decreased demand intensity.

## Discussion

In this study, we aimed to determine the role of dopaminergic neurotransmission in the relationship between price and consumption of sucrose. In contrast to our predictions, chemogenetic activation of VTA dopamine neurons increased demand elasticity, reflecting an increased sensitivity to price elevations and thus a reduced essential value. When assessing demand at a population level, we also observed a decrease in demand intensity upon VTA dopamine neuron activation. At the same time, chemogenetic VTA dopamine neuron activation increased responding for sucrose under a PR schedule of reinforcement, which is indicative of an increased incentive motivation, consistent with previous chemogenetic (Boekhoudt et al. 2018; Boender et al. 2014; Verharen et al. 2018) and pharmacological studies (Baldo and Kelley 2007; Salamone and Correa 2012). Treatment with D-amphetamine partially replicated the effects of chemogenetic mesocorticolimbic dopamine neuron activation, whereas treatment with alpha-flupentixol reduced free consumption of sucrose and had mixed effects on demand elasticity (for a summary of the data, see Table 1). Together, these findings imply that mesocorticolimbic dopamine signalling differentially influences distinct components of cost expenditure processes aimed at obtaining rewards.

**Table 1:** Summary of the results. Arrows represent a significant increase (↑) or decrease (↓) upon chemogenetic activation of mesocorticolimbic dopamine neurons or upon pharmacological dopamine manipulation. = represents no significant difference between drug and vehicle. ‘individual’ refers to the analysis of individual demand curves, ‘population’ refers to the analysis of population demand curves. *Incr.* increasing, *Decr.* decreasing, *α* demand elasticity, *Q*_*0*_ demand intensity, *ND* not determined

		VTA DA activation			D-amphetamine			flupentixol		
		# rewards	*α*	*Q*_*0*_	# rewards	*α*	*Q*_*0*_	# rewards	*α*	*Q*_*0*_
**Incr** **ratio**	Individual	↓	↑	=	↓	↑	=	↓	↑	↓
	Population		↑	↓		↑	↓		=	=
**Decr** **ratio**	Individual	↓	↑	=	↓	=	=	↓	=	↓
	Population		↑	↓		=	=		=	↓
**FR 60**		↓			ND			ND		
**PR**		↑			ND			ND		

### Contrasting effects of chemogenetic VTA dopamine neuron activation on responding for sucrose under different schedules of reinforcement

At first glance, the observed effects of chemogenetic activation of VTA dopamine neurons on responding for sucrose under the WS-IR/WS-DR and the PR schedules of reinforcement seem paradoxical. Under the WS-IR/WS-DR schedules, responding for sucrose decreased, whereas it increased under the PR schedule after VTA dopamine neuron activation. Demand analyses hence revealed that stimulation of VTA dopamine neurons reduced essential value, while incentive motivation was increased in the PR task. The observation that increased demand elasticity and increased incentive motivation occur concurrently suggests that these measures reflect different aspects of reward seeking behaviour. Indeed, contrasting outcomes for essential value and PR breakpoints have previously been reported in preclinical studies. That is, treatment with Δ9-tetrahydrocannabinol has been reported to increase the essential value of nicotine without affecting breakpoint under a PR schedule in rats, and mice lacking the serotonin transporter gene display lower PR breakpoints but similar essential value of alcohol compared to wild-type mice (Lamb and Daws 2013; Panlilio et al. 2013).

The present data demonstrate that increases in mesocorticolimbic dopamine activity do not invariably result in increased appetitive behaviour, even under high response ratio requirements. These different effects of VTA dopamine neuronal activation on responding for sucrose have to be viewed in light of the inherent differences in the schedules of reinforcement used. Demand curve procedures allow for varying levels of reward obtainment across multiple ratios on a continuous scale. Conversely, reward obtainment under a PR schedule of reinforcement is binary: either a reward is obtained or not at a certain response ratio, whereby the session — i.e. the opportunity to gain further rewards — ends in case of non-reward. As such, essential value reflects sensitivity to price changes, while the breakpoint under a PR schedule is generally thought to reflect incentive motivation, i.e. the willingness to work for reward. It has to be borne in mind, however, that increases in responding under a PR schedule may also be the result of resistance to extinction (Kearns et al. 2016), reduced sensitivity to a declining rate of reinforcement (Verharen et al. 2018) and increased action initiation (Boekhoudt et al. 2018). In fact, the latter two have been suggested to be the consequence of increased mesocorticolimbic dopamine signalling (Boekhoudt et al. 2018; Verharen et al. 2018; see below). Thus, although responding under both schedules is related to reward seeking, the essential value and PR breakpoint likely represent distinct behavioural endpoints. Moreover, the behavioural components underlying essential value and breakpoint seem dissociable in terms of their dependence on mesocorticolimbic dopamine signalling. While in agreement with earlier studies on the role of forebrain dopamine in incentive motivation (for reviews see Baldo and Kelley 2007; Salamone and Correa 2012), the present findings imply that dopamine signalling differentially affects essential value.

The current study adds to an extensive body of literature on the role of mesocorticolimbic dopamine in reward-directed behaviour. An influential theory of reinforcement learning posits that VTA dopamine neurons encode a reward prediction error (RPE), i.e. the discrepancy between anticipated and experienced reward: VTA dopamine neuronal activity increases when an experienced reward is better than expected, whereas VTA dopamine neuronal activity decreases when an experienced reward is less than anticipated. These VTA dopamine-mediated value signals are thought to serve as teaching events to steer future appetitive behaviour (Bayer & Glimcher, 2005; Schultz et al. 1997; Schultz, 2016). Consistent with this notion, inducing a positive VTA dopamine RPE signal by optogenetic excitation at the moment of reward delivery has been shown to increase conditioned reward-seeking (Steinberg et al. 2013) and to make rats less sensitive to increases in response requirement (Schelp et al. 2017). Recent work by Mohebi et al. (2019) suggests that dopaminergic modulation of motivational processes may be more complex. They reported that dopamine release in the NAc core correlates with reward expectation, while firing rates of VTA dopamine neurons did not vary with changing reward probabilities, suggesting that dopamine release may be modulated locally at the level of the NAc core, independent of the activity of VTA dopamine neurons. Moreover, these findings suggest that differential dopaminergic mechanisms may be involved in motivation for rewards, depending on variation in reward probability or price. Importantly, in the present study, VTA dopamine neuron activity was chemogenetically increased throughout the session, rather than during specific task events. Therefore, as a result of the tonic elevation in mesocorticolimbic dopamine, the phasic increases in dopamine activity during reward delivery may have been blunted, resulting in a relative reduction in sucrose reward valuation (see Verharen et al. 2018). Consequently, response levels during the WS-IR/WS-DR sessions declined, as the sucrose earned was sensed as less valuable to the animals, causing an increase in demand elasticity. It may perhaps seem counterintuitive then that chemogenetic stimulation of VTA dopamine neurons increased responding for a less valued reward under the PR schedule of reinforcement. Consistent with the present study, we have recently observed that chemogenetically enhancing the activity of VTA dopamine cells (Boekhoudt et al. 2018; Boender et al. 2014) or the VTA-nucleus accumbens projection (Verharen et al. 2018) increases responding for sucrose under the PR schedule of reinforcement. Our detailed analysis of the animals’ behaviour in those studies suggested that chemogenetic stimulation of VTA dopamine cells increases action initiation (Boekhoudt et al. 2018; see also Syed et al. 2016) and that stimulation of the VTA-nucleus accumbens pathway reduced the animals’ ability to use negative feedback to adjust subsequent behaviour (Verharen et al. 2018). Both mechanisms can contribute to increased responding under a PR schedule, in which only one reward — albeit perhaps less valued — can be earned under each ratio, and cessation of responding ends the opportunity to earn any more rewards. Thus, increased action initiation, required to resume responding after a ratio requirement has been met and the reward has been consumed, and reduced sensitivity to negative feedback, whereby the relative value of a single lever press response declines as the ratio requirement rises, could then supersede the relative decline in the subjective value of the sucrose reward, resulting in increased PR breakpoints.

**Fig. 8:**
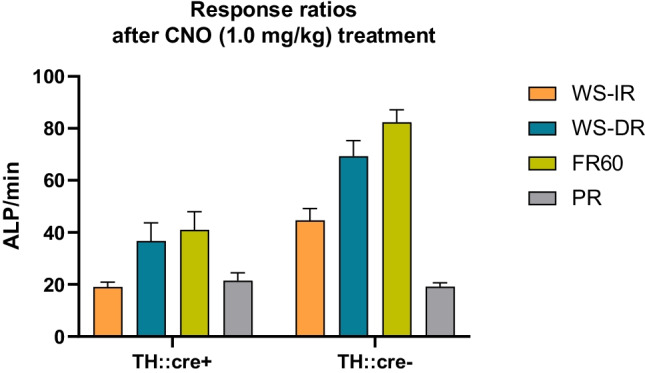
The effects of 1.0 mg/kg CNO treatment on response ratios under different schedules of reinforcement. Shown are the active lever presses (ALP) per minute under a within session increasing ratio (WS-IR) schedule, a within session decreasing ratio (WS-DR) schedule, fixed ratio (FR) 60 schedule and progressive ratio (PR) schedule of reinforcement in experimental groups I (TH::cre +) and II (TH::cre-)

Several alternative explanations should also be considered. Dopamine has been implicated in behavioural flexibility (Cools et al. 2009; Floresco 2013; Izquierdo et al. 2016; Verharen et al. 2018, 2019). Indeed, we recently showed that hyperactivity of the mesoaccumbens pathway leads to impaired flexible decision-making through interference with negative RPE processing (Verharen et al. 2018). However, we think that impaired flexibility does not play a major role in the findings in the present study, as VTA dopamine neuron activation also decreased responding throughout the FR 60 sessions, in which the response requirement was high but behavioural flexibility was not taxed. Also, impaired time perception may have interfered with task performance, since dopaminergic neurotransmission has been implicated in time perception (Lewis & Miall, 2006; Marinho et al., 2018), whereby chemogenetic stimulation of midbrain dopamine cells slows down the estimation of time (Soares et al. 2016). This could have differentially altered behaviour in the PR session, in which the rats have 30 min to obtain a subsequent reward versus the WS-IR/WS-DR sessions, in which the animals respond within a restricted 8-min time period per ratio requirement, regardless of the number of rewards obtained. However, the importance of dopamine — and perhaps dopamine-mediated RPEs —for time perception seems to involve the nigrostriatal, rather than the mesocorticolimbic dopamine system (Jahanshahi et al. 2006; Soares et al. 2016; Toren et al. 2020), making it unlikely that distorted time perception explains the effects of chemogenetic VTA dopamine neuron stimulation on responding for sucrose observed here. Also, impaired attention, which we have recently observed after chemogenetic stimulation of VTA dopamine neurons (Boekhoudt et al., 2017b), may have altered task performance. However, in this case also, the effects on attention in the 5-choice serial reaction time task were most pronounced after stimulation of substantia nigra — rather than VTA — dopamine neurons, and it would be hard to conceive how impaired attention would lead to opposite changes in responding for sucrose under the PR versus WS-IR/WS-DR schedules of reinforcement. Moreover, response rate data indicate that the findings in this study are unlikely explained by unspecific effects, such as motor fatigue or stereotypies. The response rates in the WR-IR, WS-DR and FR60 sessions of 1.0 mg/kg CNO-treated TH::cre + rats were comparable or even higher when compared to response rates in the PR sessions (Fig. 8). This suggests profound task engagement in the WR-IR and WS-DR tasks and renders it unlikely that competitive or stereotyped behaviour contributed to the lower lever press rates observed under these schedules of reinforcement. Last, satiety may have influenced task performance. Both increasing and decreasing ratio schedules were used, and animals were mildly food restricted to minimise effects of satiety and to enhance the motivation for food (Yang et al., 2020). Food restriction has been shown to alter dopamine neurotransmission (Avena et al., 2008; Sevak et al., 2008). However, a pilot study with chemogenetic activation of VTA dopamine neurons and similar WS-IR and WS-DR schedules of reinforcement for non-food deprived rats (data not shown) revealed overall lower responding, but similar effects of CNO treatment in TH::cre + animals. Therefore, it is not likely that food restriction contributed to the effects of CNO on demand and PR analyses.

### D-amphetamine and flupentixol treatment

Besides chemogenetics, pharmacological modulation of dopamine neurotransmission was used to gauge the role of dopamine in cost–benefit trade-offs for sucrose. Similar to the effects of chemogenetic stimulation, D-amphetamine decreased responding for sucrose under the WS-IR and WS-DR schedules of reinforcement and increased demand elasticity under WS-IR conditions, reflecting an increased sensitivity to price elevations and a reduced essential value. However, there were also differences between the effects of D-amphetamine and chemogenetic VTA dopamine neuron activation on responding for sucrose. That is, the effects of D-amphetamine on demand somewhat varied depending on the schedule employed. Treatment with D-amphetamine reduced responding for sucrose and increased demand elasticity under the WS-IR schedule, which is similar to the effects of chemogenetic activation. However, D-amphetamine did not affect demand elasticity under the WS-DR schedule, since the suppressing effect of D-amphetamine on the number of rewards obtained was constant across the various ratio requirements. By contrast, treatment with the dopamine receptor antagonist alpha-flupentixol reduced the number of rewards obtained in the WS-IR and WS-DR tasks. An increase in demand elasticity was detected once, whereas lower demand intensity was found in three out of four analyses.

In general terms, these results support our chemogenetic data that adaptive cost–benefit decision making depends upon dopaminergic neurotransmission (see also Verharen et al. 2018). However, the dynamics of responding under progressive ratio schedules of reinforcement seem to be different from responding under high FR schedules. While dopamine activation reduces performance under high FR schedules of reinforcement, several studies showed increased responding for food rewards under PR schedules of reinforcement, and concurrent reductions in food intake, upon treatment with dopamine transporter inhibitors, e.g. MRZ-9547 (Sommer et al. 2014), bupropion (Randall et al. 2015), lisdexamfetamine (Yohn et al. 2016a), GBR12909 (Yohn et al. 2016b), PRX14040 (Yohn et al. 2016c), CE-123 and CD-158 (Rotolo et al. 2019, 2020) and CT-005404 (Rotolo et al. 2021). The discrepancy between the impact of dopamine activation, either through chemogenetics or by pharmacological means, on responding under PR and WS-IR/WS-DR schedules of reinforcement suggest that PR schedules of reinforcement may be less susceptible to classic rate dependency, where increases in dopamine are paralleled by reductions in response rates, perhaps in part explaining the discrepancies observed between PR and WS-IR/WS-DR schedules of reinforcement. Clearly, however, the relationship between dopamine signalling and value-based decision-making is not straightforward. In fact, the similarities in the effects of D-amphetamine and alpha-flupenthixol suggest that cost–benefit decision-making requires dopamine activity to be at an optimum and that deviations from this optimum lead to impairments in decision-making. Whether the effects of chemogenetic and pharmacological manipulation of dopamine signalling on cost–benefit decision-making result from comparable behavioural mechanisms remains to be investigated, however. Thus, the effects of alpha-flupenthixol are in line with previous studies that reported reductions in the motivation to exert effort for food rewards upon suppression of dopaminergic neurotransmission (Aberman et al. 1998; Aberman and Salamone 1999; Caul and Brindle 2001; Correa et al. 2020; Reilly 1999), whereby it is remarkable that in the present study, alpha-flupenthixol treatment affected demand intensity more than demand elasticity. However, these data need to be interpreted with caution, as only a single dose of alpha-flupenthixol was analysed, because treatment with the higher dose reduced responding to such a degree that behavioural economic analysis was not possible. In addition, there are mechanistic differences between chemogenetic stimulation of VTA dopamine neurons and D-amphetamine treatment. D-Amphetamine elevates extracellular dopamine concentrations in a largely impulse-independent manner by acting as a false substrate on the dopamine transporter (Calipari and Ferris 2013; Carboni et al. 1989; Jones et al. 1998), whereas the designer receptor hM3Dq is a G-protein-coupled receptor (GPCR), activation of which induces intracellular calcium release, thereby enhancing neuronal firing. As a result, the net effects of D-amphetamine on extracellular dopamine levels are much larger (Calipari and Ferris 2013; Carboni et al. 1989; Jones et al. 1998; Verharen et al. 2018). Moreover, in addition to dopaminergic signalling, other neurotransmitter systems are affected by D-amphetamine and flupentixol. D-Amphetamine affects noradrenaline and serotonin signalling, and flupentixol is thought to have antagonistic properties at serotonin receptors as well (Leysen et al. 1993; Meltzer et al. 1989; Pum et al. 2007; Rothman et al. 2001; Sloviter et al. 1978; Soyka and De Vry 2000). The contribution of these divergent neurochemical effects to the observed differences between the effects of pharmacological and chemogenetic manipulations of dopamine function on cost–benefit decision-making warrants further investigation.

### Strength and limitation

A strength of the current study is the within-session approach, which enabled us to derive demand curves from single experimental sessions. In preclinical behavioural studies, demand curves are typically derived from between-sessions approaches through series of daily sessions that each determine the demand at one specific price. However, this is time-consuming and may hamper reliable testing as neural manipulations have to be repeated several times (Bentzley et al. 2013; Oleson and Roberts 2019).

A limitation is that dopamine activity was chemogenetically increased throughout the forebrain, including the striatum, prefrontal cortex and amygdala, and that pharmacological treatment was systemic. Therefore, there is a lack of specificity to discern the effects of dopamine on distinct brain regions regarding cost–benefit trade-offs. Especially dopamine signalling in the nucleus accumbens has been widely implicated in effort-based choice behaviour, but likely not exclusively because dopamine terminal release in the nucleus accumbens has been shown to lead to similar results as VTA dopamine stimulation on demand, but the accumbal stimulation effects were weaker (Schelp et al. 2017). Moreover, optically enhanced dopamine in the nucleus accumbens was sufficient to shift preference towards a choice with higher costs (i.e. a longer delay), but did not alter preference to a lower benefit (i.e. a smaller reward magnitude; Saddoris et al. 2015). This suggests that dopamine signalling in other brain regions than the nucleus accumbens contributes to effects on demand and that dopamine signalling in different brain regions might differentially affect separate aspects of cost–benefit balances.

## Conclusion

To conclude, these findings show that chemogenetic stimulation of dopaminergic neurotransmission altered cost–benefit decision making in a complex manner. It reduced the essential value of palatable food, increased sensitivity to price elevations and increased incentive motivation, while leaving free consumption unaltered. Together, these data extend the notion that aberrant dopamine signalling might underlie deficits in cost–benefit trade-offs in a complex manner as seen in several psychiatric disorders. Future research into the process of cost–benefit assessment and the relative contribution of distinct aspects of reward valuation will be needed to comprehend how forebrain dopamine neurotransmission precisely contributes to value-based decision-making.

## Conflict of interest

The authors declare no competing interests.

## Supplementary Information

Below is the link to the electronic supplementary material.

Supplementary file1 (PDF 4 KB)

Supplementary file2 (PDF 4 KB)

Supplementary file3 (PDF 350 KB)
