# Supplementary figures and images for: Increased elasticity of sucrose demand during hyperdopaminergic states in rats
Source: Psychopharmacology (Berl). 2022 Jan 31;239(3):773–94. doi: 10.1007/s00213-022-06068-x (PMC8891210; doi:10.1007/s00213-022-06068-x)

**Response ratios  
after CNO (1.0 mg/kg) treatment**

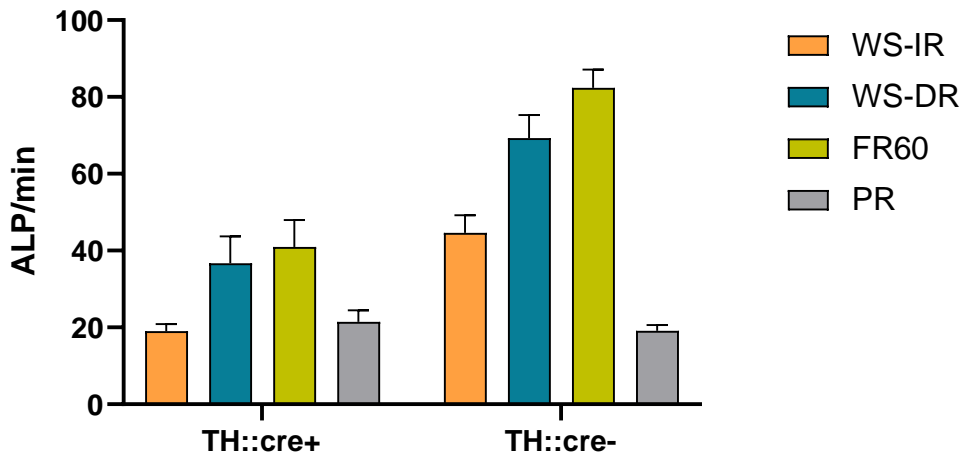

Supplement: Supplementary file 1 — Supplementary file1 (PDF 4 KB) [file 213_2022_6068_MOESM1_ESM.pdf]
